# Editorial: Metabolic dysregulation in neurodegenerative diseases

**DOI:** 10.3389/fncel.2025.1591313

**Published:** 2025-04-01

**Authors:** Maria Jimenez-Gonzalez

**Affiliations:** Diabetes, Obesity and Metabolism Institute, Icahn School of Medicine at Mount Sinai, New York, NY, United States

**Keywords:** brain, neurodegeneration, metabolism, mitochondria, glucose

The increasing prevalence of metabolic diseases such as metabolic syndrome, diabetes, and obesity appears closely linked to the rise of neurodegenerative disorders, including Alzheimer's and Parkinson's disease (Procaccini et al., 2016). Both clinical and preclinical research strongly support this connection, underscoring the urgent need for deeper investigation. Advancing our understanding of the metabolism-neurodegeneration link is crucial for developing more effective therapies and diagnostic tools.

This editorial provides a concise overview of the recently published Research Topic, *Metabolic dysregulation in neurodegenerative diseases*. This Research Topic includes four original research articles and two reviews, shedding light on key aspects of glucose metabolism in the brain, mitochondrial dysfunction, and other mechanisms contributing to neurodegeneration in the context of metabolic disorders.

## Chronic hyperglycemia, mitochondrial alterations, and mTOR pathways

Jodeiri Farshbaf et al., conducted a comprehensive study on the effects of chronic hyperglycemia in the medial habenula and interpeduncular nucleus—brain regions linked to mood disorders, addiction, anxiety, and diabetes in smokers. Using a mouse model, they identified early, transient changes in mitochondrial morphology and an increase in mitochondrial numbers in the medial habenula, which normalized over time. Additionally, they observed alterations in neural lipid composition in the interpeduncular nucleus. These findings highlight the importance of studying long-term metabolic impairments, revealing a dynamic response in brain regions critical for mood and behavior.

One of the most studied molecular pathways in Alzheimer's disease (AD) is the mTOR signaling pathway, which is vital for neuronal survival and function. Since mTOR is activated through insulin/IGF signaling, evidence suggests that diabetes and insulin resistance contribute to its dysregulation. Using a rat model of sporadic AD, de la Monte and Tong, examined behavioral and cognitive impairments alongside histological changes in the temporal lobe. They also identified molecular alterations related to AD markers, neuroinflammation, oxidative stress, and mTOR signaling. These findings reinforce the significance of IGF/insulin/mTOR signaling and highlight the need for further research into this pathway as a potential therapeutic target.

## Repurposing antidiabetic treatments

Multiple sclerosis (MS) is a complex neurodegenerative disease with a rising prevalence. As an autoimmune disorder that damages myelin-producing cells, most current therapies focus on immunosuppression. However, impaired glucose metabolism is frequently observed in MS patients (Mathur et al., 2014).

Metformin, a widely used diabetes drug, has shown promise in promoting myelin repair in preclinical models and is currently being investigated in clinical trials. It activates AMPK, a key regulator of energy metabolism, yet its effects on oligodendrocytes—the cells responsible for myelin production—and their precursors remain poorly understood.

Narine et al., explored metformin's influence on glucose metabolism and oligodendrocyte progenitor differentiation in young mice, as well as its effects in a chemical model of demyelination. Their study found that metformin significantly alters cellular metabolism and enhances the differentiation of precursors into oligodendrocytes, potentially improving myelin repair and function.

## Insights from rare diseases and genetic variants

Neurodegenerative diseases encompass a wide spectrum of disorders, many of which are driven by single pathogenic variants affecting neuronal energy production and mitochondrial metabolism. Studying these genetic mutations provides valuable insights into how glucose metabolism influences neurodegeneration.

One of the studies in this topic characterized a novel pathogenic variant of the *ACO2* gene, which encodes aconitase 2, a mitochondrial enzyme essential for the TCA cycle and mitochondrial DNA integrity. Mutations in ACO2 have been linked to severe neuropathologies, including infantile cerebellar retinal degeneration. Yang et al., identified new ACO2 variants in an infant patient with cerebellar retinal degeneration. Their analysis revealed compound heterozygous mutations, c.854A>G (p.Asn285Ser) and c.1183C>T (p.Arg395Cys), alongside alterations in metabolic, immune, and neurophysiological pathways. These findings suggest that ACO2 mutations impact more than just mitochondrial function, further highlighting their relevance to neurodegenerative processes.

Another work presented in this topic, a thorough review by Santucci et al., examined the role of impaired brain glucose metabolism in Progressive Myoclonus Epilepsies (PMEs)—a group of rare inherited neurological disorders that lead to myoclonus jerks, seizures, and progressive neurological decline. Specifically, the review focuses on the subgroup of neuronal ceroid lipofuscinoses (NCLs), where mutations disrupt genes essential for proper lysosomal function. The authors discuss glucose hypometabolism as a hallmark of the disease and explore the potential of antidiabetic drugs like metformin and GLP-1 analogs in slowing neurodegeneration. Their review highlights the therapeutic potential of these treatments for PMEs, emphasizing the need for further research.

## Vascular interactions and the blood-brain barrier

Brain energy metabolism is profoundly influenced by alterations in the blood-brain barrier (BBB), which regulates the transport of nutrients, hormones, and other essential factors. Notably, reduced BBB integrity is frequently observed in Alzheimer's (AD) and Parkinson's disease (PD), contributing to cognitive decline.

Lv et al., provide a comprehensive review of the role of platelets in increasing BBB permeability in the context of brain pathology. Their analysis sheds light on how vascular dysfunction contributes to neurodegeneration, emphasizing the importance of further investigation into BBB integrity and its therapeutic implications.

## Conclusion

The studies compiled in this Research Topic highlight the intricate relationship between metabolic dysfunction and neurodegeneration. They emphasize the need for an interdisciplinary approach to further explore the underlying mechanisms of this interaction and develop more targeted therapies, including the repurposing of existing metabolic disease treatments.
